# Thyroid Hormone Diseases and Osteoporosis

**DOI:** 10.3390/jcm9041034

**Published:** 2020-04-06

**Authors:** Alessandro P. Delitala, Angelo Scuteri, Carlo Doria

**Affiliations:** Department of Clinical and Experimental Medicine, University of Sassari, 07100 Sassari, Italy; d341elefante@virgilio.it (A.S.); cdoria@uniss.it (C.D.)

**Keywords:** thyroid dysfunctions, subclinical hypothyroidism, subclinical hyperthyroidism, osteoporosis, bone mass density

## Abstract

Thyroid hormones are essential for normal skeletal development and normal bone metabolism in adults but can have detrimental effects on bone structures in states of thyroid dysfunction. Untreated severe hyperthyroidism influences the degree of bone mass and increases the probability of high bone turnover osteoporosis. Subclinical hyperthyroidism, defined as low thyrotropin (TSH) and free hormones within the reference range, is a subtler disease, often asymptomatic, and the diagnosis is incidentally made during screening exams. However, more recent data suggest that this clinical condition may affect bone metabolism resulting in decreased bone mineral density (BMD) and increased risk of fracture, particularly in postmenopausal women. The main causes of exogenous subclinical hyperthyroidism are inappropriate replacement dose of thyroxin and TSH suppressive L-thyroxine doses in the therapy of benign thyroid nodules and thyroid carcinoma. Available data similarly suggest that a long-term TSH suppressive dose of thyroxin may decrease BMD and may induce an increased risk of fracture. These effects are particularly observed in postmenopausal women but are less evident in premenopausal women. Overt hypothyroidism is known to lower bone turnover by reducing both osteoclastic bone resorption and osteoblastic activity. These changes in bone metabolism would result in an increase in bone mineralization. At the moment, there are no clear data that demonstrate any relationship between BMD in adults and hypothyroidism. Despite these clinical evidences, the cellular and molecular actions of thyroid hormones on bone structures are not complete clear.

## 1. Introduction

Osteoporosis is by far the most common metabolic bone disease and is characterized by low bone mass with microarchitectural alteration of bone structure, leading to reduced bone strength, which predisposes to an increased fracture risk. The definition of osteoporosis is based on densitometric measurement of bone mass in the spine or hip and not on clinical criteria. The World Health Organization defines osteoporosis as a bone mineral density (BMD) 2.5 or more standard deviations (SDs) below that of a young adult at any site (T score), whereas osteopenia is defined when BMD is >1 SD and <2.5 SD lower than the young- adult mean end [1].

Decreased bone mass and increased fragility in age-related osteoporosis can occur because of failure to achieve optimal peak bone mass. Although genetic determinants are responsible for up to 85% of the variation in peak bone mass, other factors during childhood and adolescence can affect the ability to achieve optimal peak bone mass. These variables include nutrition, particularly calcium intake, delayed puberty, physical activity, a wide variety of intercurrent illnesses, and social factors such as low family income [2].

Peak bone mass is usually achieved at the end of the second decade. The progressive bone loss is due to an increased bone resorption, which is the major mechanism for increased bone fragility. Any increase in the number of resorption sites produces decreased bone mass together with changes in skeletal microarchitecture, since the mechanism of resorption requires a much shorter time than that of bone formation in the bone remodeling cycle. Thus, the amount of bone formation decreases with age, leading to a more fragile skeleton. Although many variables may be involved in this process, decrease in bone formation may also be due to an age-related decline in skeletal growth factors [3]. Postmenopausal osteoporosis is a function of aging and estrogen deficiency. It is estimated that about 75% of bone loss during the first 15 years after menopause is attributed to estrogen deficiency rather than to aging [4]. The bone loss is more evident in vertebral bodies since the trabecular component is metabolically very active and decreases significantly when estrogen is deficient. The peak bone mass achieved at maturity and the age of menopause are strong predictors of an increased risk for fracture. Bisphosphonates are the cornerstone for the treatment of osteoporosis [5] and are commonly prescribed for its treatment, both in women and men [6]. More recently, other drugs (i.e., teriparatide and denosumab) are often prescribed and showed a safety profile [7], even when prescribed for therapy of non-unions [8]. However, a surgical approach sometimes necessary, in particular, in the case of vertebral compression fractures [9].

Thyroid hormones have multiple effects on body homeostasis [10,11] and metabolism [12], and their increased concentration may lead to different complications, mainly at the cardiovascular level [13]. Thyroid hormones are also essential for skeletal maturation and have an important physiological role in the maintenance of adult bone structure and strength. Although thyroid dysfunction has been known to represent a risk factor for bone disease, the role of thyroid hormone excess or deficiency in the pathogenesis of osteoporosis and risk factors of fractures has been underestimated, and the underlying mechanisms are still uncertain.

In this review, we summarize the systemic and local effects of thyroid hormone action and the bone consequence of long-term effects of thyroid dysfunction including the subclinical forms of these diseases.

## 2. Materials and Methods

We performed on PubMed a literature search for the articles published until January 2020 by using the search terms “subclinical hyperthyroidism”, “osteoporosis”, “bone mass density”, and “subclinical hypothyroidism”. Titles of interest were further reviewed by abstract.

## 3. Thyroid Hormone Physiology

The main thyroid hormones in humans are thyroxine (T4) and 3,5,3′-triiodo-l-thyronine (T3). The synthesis and secretion of these hormones are finely regulated by the thyroid-stimulating hormone (TSH) axis, whose activity is negatively regulated by thyroid hormones and cytokines [14]. TSH acts directly on the TSH receptor (TSH-R) expressed on the thyroid follicular cell basolateral membrane [15]. The nuclear genomic effect of thyroid hormones is mediated by the intracellular binding of T3 to nuclear receptor where it activates either thyroid hormone receptor α (TRα) or β (TRβ). TRs act as a hormone-dependent transcription factor that mediates transcriptional repression in the unliganded state. T3 binding results in the dissociation of co-repressors and the recruitment of co-activators resulting in stimulation of gene transcription [16]. Both TRα and TRβ are widely expressed in a tissue-specific manner. TRβ is the main receptor expressed in the pituitary and hypothalamus where it mediates the negative feedback control of the hypothalamic–pituitary–thyroid axis [17], whereas TRα is expressed in higher concentration than TRβ in the skeleton, where it mediates T3 action on bone and cartilage [18]. The knockout of the thyroid hormone TRα gene results in delayed bone maturation, while the lack of all isoforms of TRβ has no effect on bone cells [18].

The relation between thyroid hormones and bone metabolism is well documented in children with hypothyroidism. In severe untreated hypothyroidism, delayed skeletal development, defective endochondral ossification, short stature, and epiphyseal dysgenesis are well known clinical features that can be reversed particularly when early and prompt replacement therapy is started. Thyroid hormone is crucial for cartilage growth and differentiation and enhances the response to growth hormone.

In adults, overt hypothyroidism induces a low bone turnover with a prolonged bone remodeling cycle, which is caused by reduced osteoclastic bone resorption together with decreased osteoblastic activity. On the contrary, in states of hyperthyroidism, there is a high bone turnover with a shortened remodeling time. The discrepancy between bone formation and resorption results in a negative balance with a net loss of bone.

Nuclear T3 receptor has been found in osteoblastic cell lines [19] as well as in osteoclastoma-derived osteoclasts [20]. Moreover, T3 directly stimulates bone resorption in vitro [21] possibly through the involvement of interleukin 6, a potent stimulator of osteoclastic activity. An additional role in bone metabolism has been proposed for TSH, since the TSH receptor, although predominantly expressed in thyroid follicular cells, has been described in other tissues including osteoblasts and osteoclasts [22]. TSH has been suggested as a key negative regulator of bone turnover, with a direct effect on osteoblastic bone resorption due to decreased local production of tumor necrosis factor-alpha [23]. However, this hypothesis did not explain the increased risk of osteoporosis reported in patients with Basedow’s disease. Indeed, these patients have increased levels of circulating TSH receptor autoantibodies that stimulate the TSH receptor. Thus, another study showed that a high dose of TSH was unable to affect the differentiation or function of both osteoblasts and osteoclasts in vitro [24]. These data would suggest that the skeletal abnormalities found in hypothyroidism are independent of systemic TSH. Studies on humans are limited to the effect of different TSH concentration on serum markers of bone metabolism. Interesting studies analyzed the effect of acute administration of recombinant human TSH (rhTSH) in patients without thyroid glands and with constant free thyroxine (FT4) level due to their L-thyroxine (L-T4) supply. This test is commonly used as a part of follow-up in patients with differentiated thyroid cancer and gives the opportunity to test in vivo the effect of increased concentration of TSH on bone turnover markers. One study reported an increase of N-terminal propeptide of type-I procollagen, an index of osteoblastic activity, and an increase of serum receptor activation for nuclear factor kB ligand (RANKL) in postmenopausal women but not in premenopausal women, suggesting a possible role of estrogen on bone reactivity to TSH [25]. On the other hand, another study reported no effect of rhTSH on serum osteoprotegerin and RANKL [26].

## 4. Bone Physiology

The skeleton is one of the largest organs in the body and has multiple physiological actions including the structural strength and integrity of the body, having an important role in the maintenance of normal serum level of calcium and phosphate. Bone consists of a mineralized matrix and a highly metabolic active cellular component. Cortical bone is a dense bone, which represents up to 80% of skeletal mass, and is present in the shafts of long bones. The bone remodeling cycle is initiated and orchestrated by osteocytes, which are embedded within mineralized bone and communicate via ramifications of dendritic processes. Bone remodeling may result from changes in mechanical load, structural damage, or exposure to systemic or paracrine factors. Hemopoietic cells of the monocyte/macrophage lineage differentiate to mature osteoclasts and resorb bone [27]. During the reversal phase, osteoblastic progenitors are recruited to the site of resorption, differentiate and synthesize osteoids, and mineralize the new bone matrix to repair the defect. Crosstalk between bone-forming osteoblasts and bone-resorbing osteoclasts regulates bone remodeling and maintains skeletal homeostasis [18].

Bone is formed by osteoblasts, which are derived from mesenchymal cells in the skeletal microenvironment; they are connected by gap junctions and secrete collagen and non-collagen proteins [28]. Mature osteoblasts may die by apoptosis or become osteocytes, which are embedded in the matrix or are transformed into flattened lining cells, which cover a large proportion of the bone surface. Although osteocytes may still synthesize collagen and other proteins, they have an important role in bone repair by providing active molecules for the initiation of bone remodeling at the site of bone damage. This effect is probably mediated by mechanical forces that produce a fluid shear stress in the canaliculi between osteocytes, thereby inducing intracellular signals, which result in the secretion of active molecules. Most of the molecules, including hormonal factors, act on osteoblasts for initiating bone resorption. In particular, osteoblasts secrete RANKL (receptor activation for nuclear factor kB ligand) and CSF1 (colony-stimulating factor 1), and other factors such as cytokines, prostaglandins, and growth factor that regulate bone resorption and are crucial for osteoclastogenesis [29]. The most important stimulator of osteoclastic formation is RANKL, a member of the Tumor Necrosis Factor (TNF) protein superfamily. All factors involved in osteoclast genesis stimulate RANKL by osteoblastic cell lines. Osteoclastic formation is inhibited by osteoprotegerin (OPG), which is a soluble receptor for RANKL, thereby preventing interaction of RANKL with its receptor RANK, a member of the TNF receptor family [30].

Resorption and formation of bone occurs throughout life and is a coordinated process that is regulated by basic multicellular units, that are activated to maintain skeletal strength and repair bone microdamage [31]. The bone remodeling cycle, which is influenced by systemic hormones and local factors, is characterized by activation, resorption, reversal, and formation steps, and this turnover is greater in cancellous than in cortical bone. In this cycle in young adults, the amount of new bone formed in the resorptive cavity is equal to the amount resorbed by osteoclasts. When the activity of osteoclasts is enhanced and/or that of osteoblasts is reduced, there is a net loss of bone with an increased risk of osteoporosis [32].

Although a direct action of T3 on osteoblasts and chondrocytes is recognized, the effect of T3 on osteoclasts is still unclear [33]. Thyroid hormones might have a direct effect on osteoclasts, or their action on bone resorption might be mediated by osteoblasts or other cell types.

The most widely used method for measuring bone mass is dual-energy X-ray absorptiometry (DXA). The technic provides accurate values for bone mineral content (BMC) and BMD in the lumbar spine, the proximal femur, the distal radius, and the whole body, with minimal radiation exposure. BMD is calculated from BMC and the area of bone scanned (g/cm^2^). Ultrasonography, particularly in the calcaneous, is a rapid procedure, which does not use X-rays and may predict fracture risk. Data of both techniques are reported in terms of T scores (i.e., standard deviations from the young adult values) or Z scores (i.e., standard deviation from the expected normal values). DXA scanners are also used for vertebral fracture assessment, which offer a high degree of accuracy in diagnosing fractures [34]. Other methods include quantitative computed tomography (QCT), which is likely more accurate than DXA.

## 5. Hyperthyroidism

The main causes of hyperthyroidism are Graves’ disease, toxic multinodular goiter, and toxic adenoma (Table 1). Toxic multinodular goiter is the most frequent cause of spontaneous hyperthyroidism in areas with a low iodine intake and hyperthyroidism may also be precipitated by excess iodine intake from drugs or radiographic contrast agents. Overt thyroid disorders, defined as a suppression of TSH with increased FT4 and/or free triiodothyronine (FT3) are clinically evident and an early diagnosis is usually made. Due to its deleterious effect on the cardiovascular system [35], treatment of hyperthyroidism is always necessary to ensure a rapid relief of symptoms and to avoid long-term consequences. For these reasons, severe long-lasting hyperthyroidism is now rarely encountered in clinical practice. It is well known that overt hyperthyroidism has a detrimental effect on bone mass and fragility fractures due to a high bone turnover as documented by a shortened bone remodeling cycle, together with an increase in biochemical markers of bone resorption and bone formation [18].

The accelerated remodeling cycle causes an increased release of calcium into the systemic circulation [36]. High levels of calcium reduce parathyroid hormone secretion leading to an increased urinary calcium loss and a negative calcium balance. The conversion of vitamin D into its active form is also reduced by low parathormone thus reducing gastrointestinal calcium absorption and resultant fecal calcium losses.

Some studies demonstrated 12–20% reduction in the BMD of hyperthyroid subjects [37], while other authors found no difference in BMD between thyrotoxic patients and euthyroid subjects [38]. A meta-analysis of 25 studies reported by Vestergaard [39] showed that BMD was decreased in untreated patients with hyperthyroidism, with an increased risk of hip fracture, which increases significantly with age. This aspect is more evident in postmenopausal women with a three-to-fourfold increase in fracture [40]. Similar results have been reported by Jodar et al. both in postmenopausal women with Graves’ disease and in those with toxic nodular goiter [41]. BMD was also evaluated in geriatric patients with toxic nodular goiter [42]. Both women and men had a BMD that was significantly decreased compared to the control. Longitudinal studies also evaluated whether bone loss could be reversed by treatment of hyperthyroidism. A study by Dhanwal et al. reported an early recovery at hip and lumbar spine after eight weeks of carbimazole therapy [43]. Another study reported that the increased level of OPG found in hyperthyroid, normalized after medical treatment, even in the presence of persistent abnormal bone structure [44]. Collectively, all the data demonstrate that the severity of hyperthyroidism appears to influence the degree of bone mass and increase the probability of osteoporosis. Moreover, the majority of studies in hyperthyroid postmenopausal women demonstrated a reduction in BMD. Furthermore, a prior history of hyperthyroidism is an independent risk factor for hip and vertebral fracture (relative risk 1.8) [45]. Data seem also to suggest that treatment of hyperthyroidism has a beneficial effect on bone metabolism, but studies are too scanty to draw firm conclusions.

## 6. Subclinical Hyperthyroidism

Subclinical disorders of the thyroid gland, defined as TSH outside the reference range of values and FT3 and FT4 within the normal range, are more subtle diseases and the diagnosis is incidentally made during screening exams. The availability of sensitive assay for TSH has allowed recognition of a syndrome in which usually there are no signs or symptoms of thyrotoxicosis, but the serum TSH is subnormal. Subclinical hyperthyroidism, defined as low TSH and free hormones within the reference range, has gained attention in the last years for its association with cardiovascular disease (CVD), in particular with atrial fibrillation in patients with a serum TSH concentration of 0.1 mU/L or less. The incidence of subclinical hyperthyroidism increases with age, especially in women, and is present in about of 15% of women over the age of 60 [46]. Serum TSH may also progress to overt hyperthyroidism, particularly, in patients with autonomous thyroid nodules or multinodular goiter. A reduction in serum TSH can occur with euthyroid Graves’ disease, subacute thyroiditis, autonomous adenoma, multinodular goiter, or administration of amounts of thyroid hormone greater than that required for metabolic needs. Exogenous hyperthyroidism is due to iatrogenic over-replacement with thyroid hormone supplementation. This condition is present when TSH suppressive therapy is prescribed in patients with differentiated carcinoma of thyroid or with benign uninodular or multinodular goiter.

## 7. Endogenous Subclinical Hyperthyroidism

The possibility that subclinical hyperthyroidism is involved in fracture risk has been investigated in a number of studies on bone turnover BMD measurement or fracture risk in pre- and postmenopausal women and in men [47], as reported in Table 2. No effect of endogenous subclinical hyperthyroidism on bone turnover was reported by Gurlek and Gedik in fifteen premenopausal women with a stable TSH suppression during a period ranging between 6 and 11 months [48]. Persistent increase in bone turnover in Graves’ patients with subclinical hyperthyroidism was observed in other studies. However, no data on bone density were studied [49]. Women older than 65 years of age who have suppressed TSH have been reported to have a threefold increased risk for hip fractures and a fourfold increased risk for vertebral fracture [41]. Decreased BMD was observed in a small group of patients with subclinical hyperthyroidism with nodular goiter [50]. Similar data have been reported in pre- and postmenopausal women with subclinical hyperthyroidism caused by multinodular goiter. BMD and bone turnover markers were decreased and respectively higher in post- than in premenopausal ones [51]. A cross-sectional study on data collected in the third U.S. National Health and Nutrition Examination Survey (NHANES III) showed an association between low serum TSH and osteopenia and osteoporosis. Interestingly, a graded increase in BMD with increased serum TSH across the normal range was observed in healthy American women of both black and white races [52]. Low bone density together with high biochemical markers of bone turnover has been confirmed in additional studies [53,54,55,56], although no association between subclinical hyperthyroidism and BMD or hip fractures in older men and women was found in a large cohort study of community-dwelling individuals 65 years old or older enrolled in the Cardiovascular Health Study [57].

In the review paper on the skeletal consequences of thyrotoxicosis by Nicholls et al. [18], the authors concluded that endogenous subclinical hyperthyroidism may be associated with increased biochemical markers of bone turnover and a small reduction in BMD. An increased risk of fracture may be present, although these alterations are particularly found in postmenopausal but not in premenopausal women [18]. Two more recent meta-analysis studies on subclinical thyroid dysfunction and fracture risk gave additional support to the clinical effects of subclinical hypothyroidism on bone metabolism [65,66]. This clinical condition was associated with an increased risk of hip and other fractures, particularly in patients with TSH <0.10 mIU/L, but not with TSH between 0.1 and 0.4 mIU/L [67] and older than 65 years, when osteoporosis per se has a high prevalence. On the other hand, a recent study did not find any association of subclinical thyroid disease with bone turnover markers or hip fractures in older men [68], whereas a greater osteoporosis risk due to accelerated hip bone loss in subclinical hyperthyroidism has been reported in a systematic literature search in MEDLINE/EMBASE (1946–016) [69].

Despite some negative results, the available data, taken together support the concept that subclinical hypothyroidism may affect bone metabolism and may be associated with an increased risk for hip and other fractures. The effect of treatment of subclinical thyroid dysfunction on BMD gave conflicting results [70], but large randomized controlled trials are needed. Recent guidelines recommend treatment of subclinical hyperthyroidism in all patients older than 65 years with TSH persistently 0.1 mIU/L or lower and in those with symptoms or comorbidities [71]. The treatment in older subjects is aimed to reduce the incidence of cardiovascular disease. However, TSH measurement should be repeated at least after 4 weeks in order to discriminate transient (in particular transient thyroiditis) and persistent subclinical hyperthyroidism and avoid overtreatment. If a suppressed TSH is confirmed, the possible increased risk of fractures associated with subclinical hyperthyroidism might benefit from this treatment, although there are no randomized studies on this topic.

## 8. Exogenous Subclinical Hyperthyroidism

The main causes of exogenous subclinical hyperthyroidism are inappropriate replacement dose of thyroxine, TSH suppressive thyroxine doses in the therapy of benign thyroid nodules, and thyroid carcinoma. Differentiated thyroid carcinoma (DTC)—papillary and follicle carcinoma—is the most frequent endocrine malignancy [72]. After an initial total thyroidectomy followed by radioactive iodine ablation of thyroid remnant and/or metastatic lesion, patients with DTC are treated with doses of L-T4 sufficient to suppress the circulating TSH concentration because it inhibits further growth of any residual neoplastic tissue and prevents progression of thyroid cancer and relapse [73]. Despite increased relapse-free survival, long-term TSH suppression represents a state of chronic subclinical hyperthyroidism and may be associated with adverse side effects on bone metabolism, cardiac function, and an increased risk of atrial fibrillation, particularly in the elderly.

Early studies reported a negative effect on bone metabolism with a reduced bone mass in patients on prolonged L-T4 treatment with reduced serum TSH [74,75,76], as reported in Table 3. However, additional studies failed to confirm these data [77,78,79]. No effect of prolonged L-T4 treatment on bone mass was observed in premenopausal women with reduced serum TSH in a meta-analysis report, whereas postmenopausal women with subclinical hyperthyroidism due to TSH-suppressive doses of L-T4 had reduced bone mass [80]. In postmenopausal women on L-T4 therapy, bone turnover is related to serum TSH concentration the reduction of L-T4 dose is beneficial to BMD and bone turnover [81], whereas L-T4 doses able to suppress TSH without inducing subclinical hyperthyroidism did not induce adverse effects on BMD [82]. Lack of effect on BMD of long-term therapy with L-T4 TSH suppressive for DTC was also reported both in premenopausal and postmenopausal women [83]. Similar data were reported by Belaya et al. [53]. Studies in young patients with exogenous subclinical hyperthyroidism have been reported to have no effect on peak bone mass [84]. A recent cross-sectional study performed in patients treated with a supraphysiological dose of L-T4, initiated during childhood or adolescence for treatment of DTC during the attainment of peak bone mass also suggests that L-T4-mediated subclinical hyperthyroidism does not have significant negative effects on BMD and microarchitecture [85]. Overall, the available evidences suggest that premenopausal women on chronic TSH suppressive treatment with L-T4 do not have adverse effect on BMD. On the contrary, postmenopausal women on TSH suppressive doses of L-T4 are at risk of bone loss, particularly, when osteopenia or osteoporosis are already present. Finally, a very recent meta-analysis study on the influence of TSH suppression on BMD in patients with DTC suggested a possible association between L-T4-mediated TSH suppression and the lower BMD in postmenopausal women, but not in premenopausal women and men [86].

## 9. Hypothyroidism

Overt hypothyroidism is defined as increased TSH together with T3 and T4 below the lower limit of the reference range. It impairs bone turnover by reducing both osteoclastic bone resorption and osteoblastic activity. Causes of overt hypothyroidism are reported in Table 4. These changes in bone metabolism would result in an increase in bone mineralization. Data on BMD and fracture risk in hypothyroidism are scanty and inconclusive. At the moment, there are no clear data that demonstrate any relationship between BMD in adults and hypothyroidism.

## 10. Subclinical Hypothyroidism

Subclinical hypothyroidism is by definition a laboratory diagnosis and is defined by an elevated TSH value in the presence of normal T4 and T3 serum concentration. The frequency of this clinical condition varies from 6.5 to 8.5 in different populations and tends to increase in older people [91]. Subclinical hypothyroidism is usually asymptomatic and does not have significant effects [92], apart from alteration in lipid metabolism [93,94,95]. The most common cause of subclinical hypothyroidism is autoimmune thyroiditis, diagnosed through the presence of circulating autoantibodies directed against antigens of the thyroid gland. No replacement therapy is needed until TSH is >10mIU/L [91]. Data on subclinical hypothyroidism effect on bone metabolism are rather scanty, as shown in Table 5. Reduced femoral BMD has been reported in subclinical hypothyroidism [55], whereas thyroid autoimmunity has been suggested to be a potential marker of higher fracture risk in these patients [96]. On the contrary, no association was found between subclinical hypothyroidism and hip fracture risk or BMD in men and women [57,58,59,61]. Thus, the available evidences cannot demonstrate the possible bone consequence of subclinical hypothyroidism in adults.

## 11. Treatment of Subclinical Thyroid Disorders

Very few studies analyzed the effect of the treatment of thyroid disorders on bone metabolism (Table 6). In one study, Faber et al. analyzed 28 postmenopausal women before and after 2 years of the treatment of subclinical hyperthyroidism with radioiodine. Authors showed that BMD at spin tended to increase after one year and the effect was stable [80] after 2 years. Similar results were found by other authors [81,97,98,99] while others failed to demonstrate a beneficial effect of the treatment of subclinical hyperthyroidism. No prospective studies have been done for patients with subclinical hypothyroidism.

## 12. Conclusions

Current evidences do not allow to draw clear conclusions because most of the studies are limited by their small sample size. However, it is reasonable to hypothesize that subclinical hyperthyroidism may be associated with increased biochemical markers of bone turnover and a small reduction in BMD, but the effective risk of increased frequency of osteoporosis and frequency of facture needs to be elucidated. The question whether treatment of subclinical hyperthyroidism might reduce the bone loss—or even increase BMD—remains unsolved due to the lack of clear evidences.

On the contrary, subclinical hypothyroidism seems to be unrelated to the bone metabolism, again for the low number of evidences currently available.

While the treatment of overt thyroid disease is mandatory due to its effect on the cardiovascular system, lipid metabolism, and metabolic consequences, the treatment of subclinical disorders are still under debate [89]. The decision of their treatment is based on different factors and the possible reduction of the BMD cannot be considered the main reason to start appropriate therapy.

## Figures and Tables

**Table 1:** Causes of thyrotoxicosis.

Hyperthyroidism (hormone overproduction)
Graves’ disease
Toxic multinodular goiter, toxic adenoma
Iodide-induced hyperthyroidism (Jod-Basedow effect)
Amiodarone-associated hyperthyroidism due to iodide release
TSH-secreting pituitary tumors
**Thyrotoxicosis (transient hormone excess)**
Autoimmune thyroiditis
Subacute thyroiditis
Drug-induced thyroiditis (amiodarone, lithium, interferon alfa, interleukin 2, thyrosine kinase inhibitors
**Exogenous thyroid hormone**
Iatrogenic over-replacement
Thyrotoxicosis factitia

**Table 2:** Studies that analyzed the effect of endogenous subclinical hyperthyroidism on lumbar spine bone mass density and femoral bone mass density.

Study	*n*	Gender	Age (Years)	Lumbar Spine BMD (g/cm^2^)	Lumbar Spine T Score	Femur BMD (g/cm^2^)	Femur T Score	Comment
Lee [58]	96	F	13.8 ± 11.1	1.162 ± 0.160	−0.04 ± 1.43	0.959 ± 0.154	−1.12 ± 1.28	No association with BMD and T score
	1320	M	46.8 ± 10.5	1.200 ± 0.165	0.25 ± 1.38	0.984 ± 0.176	0.46 ± 1.13	No association with BMD and T score
Ding [59]	47	F	74 (67–81)	0.946 ± 0.157	NA	0.746 ± 0.112 ^#^	NA	Reduced BMD at femoral neck compared to euthyroid
Rosario [60]	90	F	74 (65–82)	0.91 (0.80–1.21)	NA	1.05 (0.69–1.38) ^#^	NA	No association with BMD and T score
Saler [61]	86	F	33.2 ± 9.5	0.920 ± 0.160	−0.63 ± 1.11	0.830 ± 0.140	−0.76 ± 1.11	No association with BMD and T score
Garin [57]	50	F	73.7 ± 6.8	1040 ± 0.290	NA	0.700 ± 0.120	NA	No association with BMD and T score
	32	M	73.8 ± 6.6	1.140 ± 0.240	NA	0.950 ± 0.160	NA	No association with BMD and T score
Ahn [62]	38	F	56.0 ± 4.4	0.520 ± 0.200	−0.72 ± 1.2	NA	NA	Reduced BMD and T score compared to euthyroid
Rosario [54]	48	F pre	52.9 (35–63)	1.17 (0.92–1.43)	NA	1.04 (0.80–1.29) ^#^	NA	Reduced BMD at femoral neck compared to healthy control
		F post		0.97 (0.60–1.32)	NA	0.89 (0.69–1.13) ^#^	NA	
Lee [55]	19	F	54.3 ± 7.1	0.890 ± 0.160		0.750 ± 0.080 ^#^	NA	Reduced BMD at femoral neck compared to euthyroid
Tauchmanova [51]	30	F pre	40.9 ± 7.3	NA	−0.14 ± 0.70 ^¶^	NA	−0.51 ± 0.81^¶^	Reduced Z score compared to euthyroid
	30	F post	57.7 ± 6.8	NA	−0.37 ± 0.93 ^¶^	NA	0.05 ± 0.61^¶^	Reduced Z score compared to euthyroid
Ugur-Altun [63]	8	F	33.0 ± 5.0	NA	NA	0.921 ± 0.030 ^#^	NA	No association with BMD compared to euthyroid
Gurlek [48]	15	F	28.6 ± 5.8	1.020 ± 0.100	NA	0.790 ± 0.080 ^#^	NA	No association with BMD compared to euthyroid
Foldes [64]	13	F pre	NA	1.010 ± 0.170	NA	0.870 ± 0.150 ^#^	NA	No association with BMD compared to euthyroid
	24	F post	NA	0.840 ± 0.150	NA	0.730 ± 0.120 ^#^	NA	No association with BMD compared to euthyroid

Abbreviation: F: female; M: male; F pre: female premenopausal; F post: female postmenopausal; BMD: bone mineral density; NA: not applicable; ^#^ femur neck; ^¶^ Z score.

**Table 3:** Studies that analyzed the effect of exogenous subclinical hyperthyroidism on lumbar spine bone mass density and femoral bone mass density.

Study	*n*	Gender	Age (Years)	Lumbar Spine BMD (g/cm^2^)	Lumbar Spine T Score	Femur BMD (g/cm^2^)	Femur T Score	Comment
Moon [87]	25	F pre	45.8 ± 3.1	NA	0.29 ± 0.99	NA	0.08 ± 0.86 ^#^	No association with T score
	74	F post	61.4 ± 7.6	NA	−0.84 ± 1.22	NA	−0.97 ± 0.91 ^#^	No association with Z score
Mendonça Monteiro de Barros [85]	17	F	27.4 ± 6.4	1.204 ± 0.140	0.15 ± 1.02	0.150	0.50 ± 1.15 ^¶^	No association with BMD and Z score
				NA	NA	1.058 ± 0.170 ^#^	0.24 ± 1.13 ^#^	No association with BMD and Z score
Eftekhari [88]	22	F pre	NA	1.080 ± 0.180	NA	NA	NA	No association with BMD
	33	F post	NA	0.980 ± 0.210	NA	NA	NA	No association with BMD
	11	M	NA	1.110 ± 0.210	NA	NA	NA	No association with BMD
Reverter [83]	44	F pre	39.0 ± 9.0	1.229 ± 0.167	NA	1.032 ± 0.124 ^#^	NA	No association with BMD, T score and Z score
	44	F post	58.8 ± 9.0	1.094 ± 0.248	NA	0.927 ± 0.124 ^#^	NA	No association with BMD, T score and Z score
Appetecchia [82]	40	F pre	38.4 ± 3.8	1.030 ± 0.200	0.06 ± 1.15	1.080 ± 0.090	0.15 ± 0.70	No association with BMD, T score and Z score
	56	F post	49.7 ± 1.5	0.850 ± 0.200	−0.11 ± 0.80	0.900 ± 0.0700	−0.10 ± 0.75	No association with BMD, T score and Z score
	40	F pre	NA	1.080 ± 0.160 *	0.07 ± 0.80 *	0.940 ± 0.130 *	0.13 ± 0.80 *	No association with BMD, T score and Z score
	56	F post	NA	0.810 ± 0.600 *	−0.10 ± 0.70 *	0.800 ± 0.060 *	−0.09 ± 0.78 *	No association with BMD, T score and Z score
Poomthavorn [84]	18	F	22.4 ± 4.4	1.160 ± 0.400	NA	0.980 ± 0.250^#^	NA	No association with BMD
Kim [79]	36	F post	53(57–65)	0.997 ± 0.149	NA	NA	NA	No association with BMD
			NA	0.986 ± 0.145 *	NA	NA	NA	No association with BMD
Nuzzo [89]	40	F	41.0 ± 1.6	1.070 ± 1.030	0.52 ± 0.24	0.840 ± 0.030 ^#^	−0.03 ± 0.29 ^#^	No association with BMD and Z score
Lecomte [90]	36	F	NA	0.970 ± 0.175	NA	NA	NA	No association with BMD

Abbreviation: F: female; M: male; F pre: female premenopausal; F post: female postmenopausal; BMD: bone mineral density; NA: not applicable; ^#^ femur neck; ^¶^ Z score; * after follow-up.

**Table 4:** Causes of hypothyroidism.

Primary hypothyroidism
Acquired
Hashimoto’s thyroiditis
Postablative thyroiditis due to surgery, 131 therapy, neck irradiation for non-thyroid malignancy
Drug induced (lithium, thionamide, amiodarone, iodide)
Drug-induced thyroid destruction (tyrosine kinase inhibitor).
Cytokine induced (interferon-y, interleukin-2)
Thyroid infiltration (hemochromatosis, amyloidosis, Riedel’s struma, sarcoidosis)
Iodine deficiency (endemic goiter)
Goitrogens in foodstuffs
Congenital (NIS or pendrin mutations, organification disorders, defects in thyroglobulin synthesis or processing)
Thyroid agenesis or dysplasia
TSH receptor defects
**Central hypothyroidism**
Pituitary or hypothalamic disorders
**Dopamine infusion**
Chronic severe illness
**Resistance to thyroid hormone**
**Transient hypothyroidism**
Post subacute thyroiditis

**Table 5:** Studies that analyzed the effect of subclinical hypothyroidism on lumbar spine bone mass density and femoral bone mass density.

Study	*n*	Gender	Age (Years)	Lumbar Spine BMD (g/cm^2^)	Lumbar Spine T Score	Femur BMD (g/cm^2^)	Femur T Score	Comment
Lee [90]	1320	F	46.8–10.5	1.134 ± 0.157	−0.273–1.366	0.925–0.136	−0.404–1.129	Reduced risk of osteoporosis in postmenopausal women
	552	M	49.9–12.0	1.190 ± 0.157	0.086–1.310	1.000–0.146	0.462–1.125	No association with BMD and T score
Ding [91]	100	F	70 (67–75)	0.934 ± 0.161	NA	0.752–0.119 ^#^	NA	No association with BMD and T score
Garin [57]	418	F	73.2 ± 5.6	0.920 ± 0.240	NA	0.630 ± 0.130	NA	No association with BMD and T score
	260	M	75.5 ± 6.1	1.160 ± 0.330	NA	0.760 ± 0.160	NA	No association with BMD and T score
Lee [55]	19	F	54.2 ± 7.0	0.930 ± 0.020	NA	0.740 ± 0.120 ^#^	NA	Reduced BMD at femoral neck compared to euthyroid

Abbreviation: F: female; M: male; F pre: female premenopausal; F post: female postmenopausal; BMD: bone mineral density; NA: not applicable; ^#^ femur neck.

**Table 6:** Studies that analyzed the effect of the treatment of subclinical hyperthyroidism on lumbar spine bone mass density and femoral bone mass density.

Study	*n*	Gender	Age (Years)	Lumbar Spine BMD (g/cm^2^) PRE-Treatment	Lumbar Spine BMD (g/cm^2^) POST-Treatment	Femur BMD (g/cm^2^) PRE-Treatment	Femur BMD (g/cm^2^) POST-Treatment	Comment
Rosario [97]	36	29 F	NA	NA	+1.6% *	NA	+1.9% *	Increased BMD after treatment
Yonem [99]	20	19 F	36.1 ± 1.4	0.991 ± 0.046	0.998 ± 0.048	0.828 ± 0.038 ^#^	0.826 ± 0.042 ^#^	No benefit
Faber [100]	28	F	60 (52–68)	NA	+1.9% *	NA	NA	Increased BMD after treatment
Arata [98]	14	F	20–35	0.908 ± 0.115	1.103 ± 0.139	0.751 ± 0.075	0.905 ± 0.137	Increased BMD after treatment
Guo [81]	18	F post	NA	1.150 ± 0.210	1.170 ± 0.210	0.870 ± 0.130	0.900 ± 0.150	Increased BMD after treatment
	23	F post	NA	1.060 ± 0.0170	1.050 ± 0.170	0.850 ± 0.140	0.830 ± 0.140	Increased BMD after treatment

Data are presented as mean ± standard deviation or median (interquartile range). Abbreviations: F: female; M: male; F pre: female premenopausal; F post: female postmenopausal; BMD: bone mineral density; NA: not applicable. ^#^ femur neck; * variation from baseline levels.
